# Knowledge, attitudes and practices of traditional birth attendants in pastoralist communities of Laikipia and Samburu counties, Kenya: a cross-sectional survey

**DOI:** 10.11604/pamj.supp.2016.25.2.9983

**Published:** 2016-11-26

**Authors:** Matthew Reeve, Pamela Onyo, Josephat Nyagero, Alison Morgan, John Nduba, Michelle Kermode

**Affiliations:** 1 Nossal Institute for Global Health, University of Melbourne, Carlton 3010, VIC, Australia; 2 Amref Health Africa, Nairobi, Kenya

**Keywords:** Maternal and child health, skilled birth attendants, traditional birth attendants, health systems, pastoralist communities, Kenya

## Abstract

**Introduction:**

Current efforts to reduce maternal and newborn mortality focus on promoting institutional deliveries with skilled birth attendants (SBAs), and discouraging deliveries at home attended by traditional birth attendants (TBAs). In rural Kenya, semi-nomadic pastoralist communities are underserved by the formal health system, experience high maternal and neonatal mortality, and rely primarily on TBAs for delivery care, despite Government proscription of TBA-assisted births. This study examined the knowledge, attitude and practices of TBAs serving these communities to assess the potential for collaboration between TBAs and SBAs.

**Methods:**

A cross-sectional, interviewer-administered survey was conducted among 171 TBAs from Maasai and Samburu pastoralist communities in Laikipia and Samburu counties, Kenya, as part of a larger mixed-methods study in partnership with a local service provider.

**Results:**

BAs were relatively elderly (mean age 59.6 years), and attended an average of 5-6 deliveries per year. A minority (22.2%) had received formal training. They provided antenatal, intra-partum and post-partum care. Most TBA care was non-interventionist, but not necessarily consistent with best practice. Most had encountered birth complications, but knowledge regarding management of complications was sub-optimal. Most had previously referred at least one woman to a health facility (80.1%), were key participants in decision making to refer women (96.5%), and had been present at an institutional delivery (54.4%).

**Conclusion:**

TBAs continue to be key providers of maternal and neonatal healthcare in regions where the formal health system has poor coverage or acceptability. Strengthening existing TBA/SBA collaborations could improve both community links to the formal health system, and the quality of care provided to pastoralist women, while remaining consistent with current Government policy.

## Introduction

Nearly half of global maternal deaths in 2013 occurred in sub-Saharan Africa, mostly from preventable causes [1]. Effective management of birth-related complications is essential for reducing maternal and neonatal deaths, and the presence of a skilled birth attendant (SBA) at every birth is considered the optimal strategy for achieving this. However, physical, financial and cultural barriers to accessing SBAs, combined with an SBA workforce shortage, have held back progress in SBA coverage. The reality is that many women in low and middle income countries receive delivery care from traditional birth attendants (TBAs), who are unqualified, lay practitioners. The easy availability and high cultural acceptability of TBAs in most settings, coupled with poor accessibility to alternative providers, has long fuelled debate regarding the optimal contribution of TBAs to maternal health care and outcomes, ranging from advocacy for the provision of TBA training and delivery kits in the 1970 and 1980s, to banning TBA-assisted deliveries all together, as is the case in some countries today [2, 3].

Several reviews highlight evidence that TBAs, when trained, can contribute to maternal mortality reduction [4] and facilitate access to services [2], and that integration of TBAs with formal health systems can increase skilled birth attendance across a range of integration models [5]. This evidence, along with poor progress in scaling up SBA coverage in many countries has led, once again, to reconsideration of the TBA role in maternal and neonatal care [2]. While evidence is emerging that TBAs can be effective as part of a broader intervention package, few studies have examined the current roles and practices of TBAs when considering how they might integrate with the formal health system. Kenya's high maternal and neonatal mortality reflects the slow progress seen in much of sub-Saharan Africa. The maternal mortality ratio in Kenya was 490 deaths per 100,000 live births in 1990, which had reduced to 400 in 2013, a decrease of only 17%: the lifetime risk of maternal death in Kenya is 1 in 53 [6]. While Kenya has seen recent progress in neonatal mortality, there are still 22 neonatal deaths per 1000 live births [7]. In response to these relatively poor obstetric outcomes, the Kenyan government has deployed SBAs to the lowest-tier health facilities and have made delivery care available free of cost. Nationally, 62% of Kenyan women now deliver with an SBA [7, 8], but SBA-assisted deliveries mostly occur in urban areas; only 50% of deliveries in rural Kenya are attended by an SBA, and just 8% in some pastoralist areas of Laikipia and Samburu [7, 9]. The uptake of SBA services in poor rural communities in Kenya are inhibited by lack of transport and poor road infrastructure, financial barriers (including travel and opportunity costs), and concerns about abusive, overly invasive or culturally unacceptable treatment by SBAs [10–13]. The Government has also proscribed TBA-assisted deliveries [14]. Despite this, TBAs remain the main provider of delivery care in most rural areas; 57% of deliveries in Laikipia and Samburu Counties were assisted by a TBA in 2012 [9]. TBAs live in the same village as their clients, are well known and trusted members of the community, and are seen to provide supportive and culturally acceptable care [15–17].

The transition from TBA-assisted home deliveries to SBA-assisted deliveries in health facilities is likely to be gradual at best in the more remote areas of Kenya, and TBAs will continue to play a significant role in maternal health care for years to come. Better collaboration between TBAs and SBAs has the potential to accelerate this transition [2, 18, 19]. It is therefore important to understand the knowledge, attitudes and practices of TBAs in rural communities, in order to develop feasible, acceptable, safe models of collaborative care that capitalise on the strengths of TBAs, SBAs and their communities, and harmonise with Kenyan Government policy. This survey aimed to explore knowledge, attitudes and practices relating to maternal and neonatal care among TBAs in semi-nomadic pastoralist communities of Samburu and Laikipia counties. This survey is part of a larger mixed-methods study, intended to inform the development of a collaborative model for maternal and neonatal care involving TBAs and SBAs serving pastoralist communities.

## Methods

**Study setting:** this study was a partnership between the Nossal Institute for Global Health at the University of Melbourne, Amref Health Africa, the Mothers Union of the Anglican Church in Kenya (MUACK), and the relevant County Health Ministries. The study builds on the existing health and development projects of MUACK with pastoralist communities in Laikipia and Samburu counties, Kenya. The study sites were five group ranches in Laikipia (Chumvi, Morupusi, Makurian, Naibor, Tiamamut), and three in Samburu (Kirimon, Kisima, Longewan). Participating communities are mostly semi-nomadic; men migrate with cattle during dry seasons while women and children remain at home for most of the year caring for goats and sheep.

**Study design:** a cross-sectional survey was conducted among TBAs in Laikipia and Samburu counties in July-October 2014, as part of a larger, mixed-methods study of providers and users of maternal child health services in the region. We defined a TBA as any person who was recognised and accepted by the community as such. Prior to the survey, a community mapping exercise was undertaken to identify and enumerate all TBAs across the eight group ranches, and a total of 172 TBAs were identified.

**Data collection:** the initial design of the survey questionnaire was informed by the survey objectives, findings from the qualitative phase of the larger study [13, 17], the literature, and minutes from community consultations. The questionnaire was reviewed by field staff, translated into Maa and Kiswahili and independently back-translated to check for accuracy, and then piloted. The survey was interviewer-administered, took approximately 50 minutes to complete, and included questions regarding experience; antenatal, delivery and post-natal care; and links with the formal health sector.

**Data analysis:** the data were entered and analysed using Statistical Package for the Social Sciences (SPSS) Version 15.0. Descriptive statistics, both for the whole study population and disaggregated by county, were generated. Bivariate analysis using independent sample t tests and chi-square tests were used to compare values between counties for continuous and categorical variables respectively, with α=0.05.

**Ethics:** ethics approval was granted by the Ethics and Scientific Review Committee of Amref Health Africa in Kenya, and the Human Research Ethics Committee of The University of Melbourne, Australia. Informed consent was obtained from all participants, using written or witnessed verbal methods dependent on the participant's level of literacy.

## Results

Of the 172 TBAs identified, 171 were interviewed (121 in Laikipia county and 50 in Samburu county), with one TBA refusing consent for interview.

### Demographics

All TBAs were women aged between 24 and 85 years. The mean age was 59.6 years (median 60 years), and only eight respondents (4.6%) were aged under 40 years. Ages should be considered approximate and interpreted with caution, as they are based on the respondent's own estimate confirmed with qualitative exploration matching respondents' birth, marriage and birth of first child to notable local events with fixed dates. Two thirds of respondents (66.1%) were Maasai, and 27.5% were Samburu, with small minorities coming from other tribes such as Turkana (Table 1). Almost two thirds (64.3%) were currently married, 32.7% were widowed, and a small proportion were either divorced or had never been married. All except two of the respondents (98.8%) had children of their own: the highest number of children born to a respondent was 14, and the mean number of children was 7.3 (median 8). Only 7.0% TBAs had ever attended school.

**Table 1:** Demographic information, training and experience for traditional birth attendants from Laikipia and Samburu counties, Kenya (n=171)

Variable	Overall (n=171)n (%)	Laikipia (n=121) n (%)	Samburu (n=50) n (%)	p value
**DEMOGRAPHICS**				
***Tribe***				
*Maasai*	113 (66.1)	111 (91.7)	2 (4.0)	<0.001+++
*Samburu*	47 (27.5)	2 (1.7)	45 (90.0)	
*Other (e.g. Turkana)*	11 (6.4)	8 (6.6)	3 (6.0)	
***Marital* status**				
*Married*	110 (64.3)	67 (55.4)	43 (86.0)	<0.001+++
*Widowed, divorced, never married*	61 (35.7)	54 (44.6)	7 (14.0)	
Ever attended school	12 (7.0)	3 (2.5)	9 (18.0)	<0.001+++
**TBA EXPERIENCE AND TRAINING**				
Working in more than one village	56 (32.7)	41 (33.9)	15 (30.0%)	0.63
Received any formal training	38 (22.2)	20 (16.5)	18 (36.0)	0.005++
**Number of days since last delivery**				
*≤30 days*	50 (29.2)	38 (31.4)	12 (24.0)	0.057
*31-120 days*	58 (33.9)	45 (37.2)	13 (26.0)	
*>120 days*	63 (36.8)	38 (31.4)	25 (50.0)	
**Number of deliveries attended in last four weeks**				
*None*	122 (71.3)	83 (68.6)	39 (78.0)	0.41
*One*	28 (16.4)	23 (19.0)	5 (10.0)	
*More than one*	21 (12.2)	15 (12.4)	6 (12.0)	
**Normally given payment in cash or kind for assisting with delivery**	124 (72.5)	95 (78.5)	29 (58.0)	0.006++

+ = <0.05

++ = <0.01

+++ = <0.001

### Experience as a TBA

Respondents had worked as a TBA for an average of 23.9 years (median 24 years; range = 1-60 years). The mean age at which women began working as a TBA was 35.6 years (median 35 years; range 19-63 years). The majority worked only in their own village, but 32.7% also worked in at least one other village. Most TBAs had at least one other TBA working (although not necessarily residing) in their village, with a mean of 3.6 TBAs

per village overall. Only one-fifth of TBAs (22.2%) had received any training from a health professional. TBAs from Samburu were more likely to have received training compared to those from Laikipia (36.0% cf 16.5%; p=0.008). The most commonly reported training providers overall were nurses from local public health facilities, who accounted for over half of the training in Laikipia, although in Samburu, Caritas or Catholic missionaries were the most common source of training. Information regarding the recency, length or content of training was not collected, as respondents during piloting were generally unable to reliably recall this information. There was wide variation in the reported frequency of attending deliveries. More than two-thirds of the TBAs (70.8%) had not attended any deliveries in the previous month; 36.8% had not attended any deliveries in the previous four months; and 11.1% had not attended any deliveries in the last two years. Among those who had attended at least one delivery in the previous month, the mean attendance rate was 1.69 deliveries per month. Overall, the mean delivery attendance rate per month as measured by one month recall was 0.49 (median 0; range 0-4), and as measured by eight month recall from a fixed event was 0.41 deliveries per month, suggesting an annual average delivery attendance rate of five to six deliveries per TBA. The majority of TBAs (72.5%) were normally paid money and/or in-kind for their services. Most TBAs (61.4%) received money (an average amount of KSH 329 i.e. ≈USD 3 (median 200 KSH; range 5 KSH-2000 KSH). Some TBAs also received in-kind payments of meat (30.4%) or other commodities (7.6%) such as tobacco or sugar (Table 1).

### Antenatal care

Most TBAs (91.2%) normally had antenatal contact with women prior to assisting with their deliveries. Respondents reported a mean of 2.4 antenatal visits for each woman they cared for (median 2; range 1-6).

***Dietary advice:*** almost all TBAs (97.1%) gave advice about diet during pregnancy. The most common advice, given by 49.1% of TBAs, was that pregnant women should eat whatever they wished. Specific foods that were promoted included vegetables (35.1%), porridge (33.9%), herbs (22.8%) and a mixture of milk and water (19.3%). However, most respondents also advised against some foods including meat from sick animals (64.9%) and milk from sick animals (60.2%). Other foods that were discouraged by a minority were milk (18.7%), eggs (11.7%), bitter herbs (9.9%) and alcohol (7.0%).

***Health facility antenatal check-ups:*** almost all TBAs (95.9%) reported advising women to attend a health facility for a routine antenatal check-up. Antenatal referrals were most commonly made to local dispensaries (56.1%) and health centres (28.1%), which are the two lowest tiers in the Kenyan health system but the most geographically accessible in the study sites. One-fifth (20.5%) referred to the district or referral hospital, and the remaining 7.0% to other places such as the sub-district hospital or a mobile clinic. Respondents in Samburu were much more likely to refer to the local dispensary compared with those in Laikipia (74.0% cf 48.8%; p=0.002), and much less likely to refer to the district or referral hospital (2.0% cf 28.1%; p<0.001).

### Normal delivery practice

***Hand hygiene:*** most TBAs (93.1%) reported washing their hands before assisting at the most recent delivery attended, with higher rates in Samburu than Laikipia (100% cf 90.1%, p=0.019). A much lower proportion reported wearing hand protection (e.g. gloves, plastic bags) during their last delivery: only 42.1% overall. Use of hand protection was better in Samburu, twice that in Laikipia (66.0% cf 32.2%; p<0.001). Most TBAs (80.1%) reported washing their hands before cutting the umbilical cord at their most recent delivery; this practice was more prevalent in Samburu than in Laikipia (96.0% cf 73.6%; p=0.001). Only 6.4% of TBAs had ever put their hands inside the birth canal during labour, which was more common in Samburu than Laikipia (16.0% cf 2.5%; p=0.001).

***Delivery of placenta:*** delivery of the placenta was generally expectant, and encouragingly few TBAs routinely pulled on the umbilical cord to assist delivery of the placenta. If the placenta failed to deliver, the most common interventions were to massage the woman's belly (53.8%), induce vomiting (44.4%), and/or make a referral to a health facility (28.7%). Other less common interventions were to give the woman water to drink, pull on the cord, and give herbs.

***Cord management:*** TBAs overwhelmingly advocated cutting the umbilical cord immediately after delivering the baby (98.2%). The tool usually used for cord cutting was a new/unused razor blade (89.5%). Just under half of the respondents (42.1%) usually anointed the cord stump after cutting; most commonly with ash, oil or herbs.

### Postnatal care

***Almost all TBAs stayed with their patients for many days after delivery:*** 88.9% of respondents normally stayed a week or more after delivery; 66.1% stayed at least four weeks, and the longest stay reported was 12 weeks. On average, TBAs stayed 34.2 days after delivery (median 28 days; range 1 hour-12 weeks). All but one TBA routinely gave advice about neonatal feeding during the post-natal period. Almost all TBAs (97.1%) advised exclusive breastfeeding. In contradiction to this, nearly one in five respondents (18.7%) said they recommended babies be given substances apart from breast milk during the first few weeks of life. A large majority (93.0%) said that babies should be breastfed “immediately” or “as soon as possible” after delivery.

### Recognition and management of complications

***Recognition of danger signs during pregnancy:*** respondents were asked (unprompted) to list indications that a pregnant woman might have problems during her delivery. A large majority (92.4%) were able to identify at least one indication. The most frequently named indications were weakness (37.4%), abnormal lie of the baby (36.3%), anaemia (22.8%), a large baby (17.0%), a history of problems in a previous pregnancy (15.8%), and bleeding during pregnancy (10.5%). Respondents were also asked (unprompted) to list indications that a labouring woman was in danger; 95.3% were able to identify at least one indication. The most frequently named indications were slow progression of labour (44.4%), abnormal lie/presentation (34.5%), weakness (34.5%), excessive bleeding during labour (21.1%), a large baby (16.4%), and the woman falling unconscious (12.9%). TBAs were shown pictures depicting four complications of labour (post-partum haemorrhage, obstructed labour, maternal sepsis and birth asphyxia). If they did not recognise a complication from the picture, they were informed what it was, and asked if they had seen it before. They were then asked open-ended, unprompted questions regarding the signs, symptoms and management of each complication.

***Post-partum haemorrhage (PPH):*** almost all TBAs (94.7%) had previously seen a woman with PPH. When asked to name signs and symptoms of PPH (defined as “when a woman is bleeding so much her life is in danger”), respondents commonly mentioned bleeding which did not stop (93.0%), the woman losing consciousness (66.7%), and weakness/tiredness (55.6%). For management of PPH, 62.6% of TBAs said (unprompted) that they would refer the woman to a health facility. Other interventions commonly mentioned were feeding the woman blood (59.6%) and giving her medicinal herbs (21.2%). Of the 64 TBAs who did not mention referral in response to the unprompted question, 73.4% said they would make a referral to a facility for this condition, when asked directly about it. Among all of the TBAs who said they would refer a woman with PPH to a health facility (n=154), the most frequently reported indications for referral were bleeding which did not stop (78.4%), loss of consciousness (63.2%), weakness/tiredness (45.6%), failure of initial management (12.9%), and woman in pain (8.8%).

***Obstructed labour:*** nearly all TBAs (94.7%) said they had previously seen obstructed labour. When asked to name signs and symptoms of obstructed labour, respondents commonly mentioned long duration of labour (89.5%), abnormal lie/presentation (46.8%), the woman stopping pushing (33.3%), weakness/tiredness (26.3%), a large baby (22.2%), and a change in the shape of the woman's abdomen (12.3%). Almost all TBAs (90.1%) identified referral to a health facility as their intervention of choice for obstructed labour (unprompted). Other interventions included massaging the woman (8.8%) and giving herbs (8.2%). A small number said they would call the nurse or doctor, or make the woman walk around. Of the 15 TBAs who did not mention referral in response to the unprompted question, 73.3% said they would make a referral to a facility for this condition, when asked directly about it. Among all of the TBAs who agreed that they would refer a woman with obstructed labour to a health facility (n=164), the most frequently reported indication for referral was the length of time without progress (83.0%). Other commonly mentioned indications for referral were pain (45.6%), weakness (42.1%), woman stopping pushing (26.9%), failure of initial management by TBA (12.9%) and unconsciousness (11.7%). Most TBAs (78.4%) said they would wait a day or longer before referring a labouring woman to a health facility, and 37.1% gave waiting times of two days or longer. The mean wait time was 31.3 hours, with a median of 24 hours.

***Maternal sepsis:*** nearly all TBAs (93.6%) said they had previously seen a woman with maternal sepsis. As with other obstetric complications, the most commonly described intervention for maternal sepsis was referral to a health facility (66.1%). Slightly more than half of the TBAs (53.8%) said they would use medicinal herbs to treat maternal sepsis, and 34.5% mentioned giving food to the woman as an intervention. Of the 57 TBAs who did not mention referral in response to the unprompted question, 70.2% said they would make a referral to a facility for this condition when asked directly about it. Among all of the TBAs who agreed that they would refer a woman with maternal sepsis to a health facility (n=153), the most frequently reported indications for referral to a health facility were long duration of fever (76.0%), weakness (51.5%), inability to eat or drink (39.2%), abdominal pain or tenderness (36.8%) and inability to breastfeed (17.0%).

***Birth asphyxia:*** a majority of TBAs (82.5%) said they had previously seen a baby who did not begin breathing spontaneously at birth. The commonly reported interventions for birth asphyxia were splashing the baby with water (48.5%), holding the baby upside down (40.9%), pinching the baby (24.6%), ringing a bell near the baby (17.0%) and massaging the baby (11.7%). Only 2.3% mentioned calling a doctor or nurse (Table 2).

**Table 2:** Obstetric care provided by traditional birth attendants from Laikipia and Samburu counties, Kenya (n=171)

Variable	Overall (n=171)n (%)	Laikipia (n-121)n (%)	Samburu (n=50)n (%)	p value
**ANTENATAL CARE**				
Normally has antenatal contact with women before delivery	156 (91.2)	111 (91.7)	45 (90.0)	0.72
Ever advised a woman to attend a health facility for antenatal check-up	164 (95.9)	115 (95.0)	49 (98.0)	0.38
**DELIVERY CARE**				
Washed hands before assisting with delivery (last time recall)	159 (93.0)	109 (90.1)	50 (100)	0.019+
Wore hand protection while assisting with delivery (last time recall)	72 (42.1)	39 (32.2)	33 (66.0)	<0.001+++
Washed hands before cutting cord (last time recall)	137 (80.1)	89 (73.6)	48 (96.0)	0.001++
Ever put hands inside vagina during labour	11 (6.4)	3 (2.5)	8 (16.0)	0.001++
Normally pulls on umbilical cord to remove placenta after delivery	6 (3.5)	3 (2.5)	3 (6.0)	0.26
**How soon after delivery cord is cut**				
*Immediately*	168 (98.2)	119 (98.3)	49 (98.0)	0.88
*Before placenta is delivered*	2 (1.2)	1 (0.8)	1 (2.0)	
*After placenta is delivered*	1 (0.6)	1 (0.8)	0 (0.0)	
**Tool normally used for cutting cord**				
*New/unused razor blade*	153 (89.5)	105 (86.8)	48 (96.0)	0.08
*Other (e.g. used razor, knife & scissors)*	18 (10.5)	16 (13.2)	2 (4.0)	
Usually anoints the cord stump	72 (42.1)	54 (44.6)	18 (36.0)	0.30
**POSTNATAL CARE**				
Recommends baby be put to the breast immediately after birth	159 (93.0)	113 (93.4)	46 (92.0)	0.75
Normally advises exclusive breastfeeding	165 (97.1)	116 (96.7)	49 (98.0)	0.64
Recommends baby be given supplemental feeds in first few weeks of life	32 (18.7)	25 (20.7)	7 (14.0)	0.31
**EXPERIENCE WITH OBSTERIC COMPLICATIONS**				
Has previously seen post-partum haemorrhage (PPH)	162 (94.7)	114 (94.2)	48 (96.0)	0.64
Has previously seen obstructed labour	162 (94.7)	112 (92.6)	50 (100)	0.048+
Time TBAs would wait before referring woman with obstructed labour (among those who would refer; n=167)				
<24 hours	36 (21.6)	27 (23.1)	9 (18)	0.16
24-47 hours	69 (41.3)	51 (43.6)	18 (36)	
≥48 hours	62 (37.1)	39 (33.3)	23 (46)	
Has previously seen maternal sepsis	160 (93.6)	110 (90.9)	50 (100)	0.028+
Has previously seen birth asphyxia	141 (82.5)	97 (80.2)	44 (88.0)	0.22

+ = <0.05

++ = <0.01

+++ = <0.001

### Traditional birth attendants' relationships with health facilities

Referral during labour: four out of five respondents (80.1%) had previously referred a woman in labour to a health facility. Of those who had made at least one previous referral, 53.3% said they accompanied the woman to the health facility “always” or “most of the time”. Only 14.6% said they never accompanied their patients. The decision to take a labouring woman to a health facility almost always involved the TBA (reported by 96.5% of respondents), and was usually jointly made with the woman's husband (80.1%), and occasionally with the woman's mother-in-law (7.0%). The labouring woman herself was not mentioned as a participant in the decision-making process by any respondent.

Assisting with facility-based care: over half of the TBAs (54.4%) had been present for at least one delivery in a health facility. A much smaller proportion of the overall cohort (14.0%) had assisted with a delivery at a health facility. However, nearly three quarters of the respondents (72.0%) had provided postnatal care for a woman while she was in a health facility, and most (94.6%) had provided postnatal care for a woman after discharge following a facility-based delivery.

Impact of the Kenyan Government policy on their practice: TBAs were asked whether the Kenyan government policy prohibiting them from assisting with deliveries had altered their practices. Over one third of TBAs (35.1%) were unaware of the policy, and the remainder (64.9%) said that the policy had no impact on their practice. TBAs from Samburu were much less likely to be aware of the policy than those in Laikipia (82.0% unaware in Samburu vs 57.9% in Laikipia; p=0.003) (Table 3).

**Table 3:** Traditional birth attendants’ relationships with health facilities in Laikipia and Samburu counties, Kenya

Variable	Overall (n=171)n (%)	Laikipia (n-121)n (%)	Samburu (n=50)n (%)	p value
Ever referred a woman in labour to a health facility	137 (80.1)	93 (76.9)	44 (88.0)	0.10
Accompany women in labour to the health facility (out of those who had ever referred, n=137)				
*Always or most of the time*	73 (53.3)	45 (48.4)	28 (63.6)	0.11
*Sometimes*	44 (32.1)	33 (35.5)	11 (25.0)	
*Never*	20 (14.6)	15 (16.1)	5 (11.6)	
Has been paid for referring a woman to a facility for a delivery	5 (3.6)	4 (4.3)	1 (2.3)	0.56
Has been present at a health facility delivery	93 (54.4)	61 (50.4)	32 (64.0)	0.11
Has assisted with a health facility delivery (of those who have been present)	13 (14.0)	10 (16.4)	3 (9.4)	0.36
Has provided post-natal care in a health facility (of those who have been present)	67 (72.0)	42 (68.9)	25 (78.1)	0.35
Has provided post-natal care after discharge from a health facility	157 (94.6)	108 (93.1)	49 (98.0)	0.20
Impact of policy proscribing TBAs from assisting with deliveries				
*No impact*	111 (64.9)	70 (57.9)	41 (82.0)	0.003++
*Unaware of policy*	60 (35.1)	51 (42.1)	9 (18.0)	

+ = <0.05

++ = <0.01

+++ = <0.001

## Discussion

The TBAs in this study generally provided antenatal care, delivery services and extensive postnatal care - a breadth of practice similar to TBAs in other communities in Kenya and East Africa [15, 20, 21]. Reported antenatal care practices were generally appropriate, and the majority had referred women to SBAs for antenatal care. The small number of TBAs who advised against eating milk and eggs were probably seeking to reduce the baby's size and the risk of obstructed labour [13]. These TBAs assist with relatively few deliveries each year, which is likely to limit their technical competency, especially in relation to managing obstetric complications. Even with two decades or more of experience, the cumulative number of deliveries attended by these TBAs would be low compared to those attended by Kenyan SBAs during their first few years of training and practice. TBA delivery care was characterised by a non-interventionist approach. The TBAs rarely go beyond providing massage, food and herbs, an observation also made in other Maasai communities [22]. During qualitative interviews, several respondents commented that the role of the TBA during delivery was to hold and comfort the woman, rather than actively manage the birth [13]. As such, while obviously dangerous practices by TBAs were not commonly reported, problems could result from their failure to recognise danger signs, their inability to implement simple evidence-based interventions for complications, and delayed referral.

Recognition of obstetric emergencies, although incomplete, was reasonable given that most respondents had received no formal training. Despite the low number of deliveries attended by the TBAs each year, the majority reported having seen all of the obstetric complications they were asked about - whether this indicates a high complication rate among their clientele, response bias, or a conflation of knowledge and experience, is unclear. While referral to the health facility was the most commonly mentioned intervention in response to obstetric emergencies, it appeared that referral mostly takes place after other traditional interventions have failed. In particular, the long delays reported before referring a woman with obstructed labour (mean = 31 hours) are very concerning. Whether this delay represents a delay in deciding to refer or also includes delays in procuring appropriate transport is not known. While travel time and absence of transport are key barriers to SBA utilisation in Kenya, delaying the decision to refer is also an important factor limiting women's to access appropriate care [12, 23, 24]. Compared to the other complications, relatively few TBAs reported having seen a newborn with birth asphyxia, and the interventions they initiated were often inappropriate. Only one-tenth said that they would massage the baby, which is the most appropriate response in the absence of equipment for resuscitation. During the postnatal period, TBAs gave good breastfeeding advice (although there may be some misunderstanding of what constitutes exclusive breastfeeding) and routinely provided long-term care and support, which contrasts with the one or two days of care provided at the local health facility, and the 43% of rural Kenyan women who receive a postnatal check-up from an SBA within two days of delivery [7]. Postnatal care is often provided by TBAs even after delivery in a facility, and is a clear opportunity for collaboration between SBAs and TBAs that is compatible with current Government policy. It was clear that most TBAs are already engaging with the formal health system to some extent in antenatal, delivery and postnatal care, and are open to a much greater level of engagement. TBAs were key decision makers in the referral process at delivery, and played a role in referrals during the antenatal period, so are ideally placed to increase uptake of SBA services, and augment the quality of those services. Most had experience caring for women at health facilities, and most also cared for women after discharge from a health facility, so there are opportunities for collaboration across the whole spectrum of maternal health care. The government proscription of TBA delivery services had made little difference to TBA practices. The chief effect of proscription seems to be freezing of programs working with TBAs, limiting opportunities for promoting collaborative arrangements [25].

Despite government efforts to promote skilled birth attendance, TBAs are still the predominant providers of maternal health care in the pastoralist communities of Laikipia and Samburu counties, and among many other poor and rural communities in Kenya [7, 9]. Since the international shift away from training TBAs in delivery care, a variety of models have been proposed to enhance their relationships with the formal health system. The Kenyan National Health Policy currently conceives the TBA role as including “promotion of birth-preparedness, early identification and referrals [sic] of complications, post-natal care and registration of births” [14]. These roles for TBAs are also promoted by recent national and international commentators [2, 5, 21, 26]. A more formalised collaborative model of maternal and newborn care could allow TBAs to: accompany women to health facilities for antenatal care; support labouring women and assist SBAs at the time of delivery; advise on breastfeeding; and provide postnatal care and support. TBAs in these pastoralist communities are well placed to provide these services, but would require training, a standardized description of their role, and resourcing to do so. TBA training could include: nutrition and pregnancy; pathways for referral to health facilities during pregnancy and labour; supporting the mother and assisting the SBA during delivery; and breastfeeding and postnatal care. The SBAs would also need to be trained in the nature and scope of TBA collaboration. Successful collaborative models in other settings have focused on changing SBA attitudes to TBAs, rather than the other way round [27, 28]. A transitional model should also incorporate training in delivery care and management of common obstetric emergencies for TBAs in difficult to access geographically remote settings, until access to health facilities is improved and communities embrace them.

This study has several limitations. While these findings can be considered representative of TBAs in the study areas, the extent to which they apply to other pastoralist communities in Kenya, or to TBAs in other parts of Samburu and Laikipia counties, is unknown. Although a census of TBAs in the study sites was conducted, due to the semi-nomadic lifestyle of pastoralist communities, we may not have successfully identified all eligible TBAs.

## Conclusion

In these communities where SBA coverage is still low and TBAs remain the main provider, TBAs are well placed to contribute to a collaborative model of care that links them more formally to SBAs and the health system. A collaborative care model will promote better coverage and quality of maternal and neonatal health care, and support or even accelerate the transition to skilled birth attendance in health facilities. SBAs could benefit from collaboration through improved referrals to health centres by TBAs, increased cultural acceptability of their practice, and better post-discharge care and follow-up assisted by TBAs, while TBAs could improve their skills in antenatal and postnatal care, all of which are likely to contribute to better outcomes for women and their newborn babies. Such collaboration should consider the strengths and weaknesses in the clinical and cultural knowledge and skills of both skilled and traditional birth attendants, and both cadres would need appropriate training and support in order to formalise their relationship.

### What is known about this topic

- There is mixed evidence for the effectiveness of traditional birth attendants (TBAs) in reducing neonatal and maternal mortality;
- International policy has shifted to promotion of skilled birth attendants (SBA) and avoidance or proscription of TBAs? involvement in delivery care;
- Access to SBAs remains poor in many African nations, particularly in rural areas, and TBAs continue to provide the majority of delivery services.

### What this study adds

- TBAs in selected pastoralist communities in Samburu and Laikipia districts provided extensive antenatal, delivery and postnatal care to women in selected communities, including a substantial engagement with formal health services in terms of referrals and shared care;
- TBA care was generally non-interventionist but remains sub-optimal;
- Opportunities for collaborative models of care exist which could hasten uptake of SBA services and improve quality of care among TBAs and SBAs.

## References

[1] Kassebaum NJ, Bertozzi-Villa A, Coggeshall MS, Shackelford KA, Steiner C, Heuton KR (2014). Global, regional, and national levels and causes of maternal mortality during 1990-2013: a systematic analysis for the Global Burden of Disease Study 2013. Lancet..

[2] Sibley LM, Sipe TA (2006). Transition to Skilled Birth Attendance: is there a future role for trained traditional birth attendants. J Health Popul Nutr..

[3] Fleming JR (1994). What in the world is being done about TBAs? An overview of international and national attitudes to traditional birth attendants. Midwifery..

[4] Ray AM, Salihu H (2004). The impact of maternal mortality interventions using traditional birth attendants and village midwives. J Obstet Gynaecol..

[5] Byrne A, Morgan A (2011). How the integration of traditional birth attendants with formal health systems can increase skilled birth attendance. Int J Gynaecol Obstet..

[6] World Health Organization, UNICEF, United Nations Fund for Population Activities, World Bank, United Nations, Department of Economic and Social Affairs (2014). Trends in maternal mortality, 1990 to 2013: estimates by WHO, UNICEF, UNFPA, The World Bank estimates, and the United Nations Population Division..

[7] Kenya National Bureau of Statistics, Ministry of Health, National AIDS Control Council, Kenya Medical Research Institute, National Council for Population and Development (2015). Kenya Demographic and Health Survey 2014 - Key Indicators Report..

[8] Countdown to 2015 Kenya Country Profile 2014. Countdown to 2015 2014..

[9] Nossal Institute for Global Health, Mothers Union of the Anglican Church of Kenya, Anglican Overseas Aid (2012). The Road Less Travelled: Maternal and child health baseline survey among Maasai and Samburu nomadic pastoralist communities in Laikipia and Samburu, Kenya..

[10] Byford-Richardson L, Walker M, Muckle W, Sprague A, Fergus S, Rennicks White R (2013). Barriers to access of maternity care in Kenya: a social perspective. J Obstet Gynaecol Can..

[11] Kitui J, Lewis S, Davey G (2013). Factors influencing place of delivery for women in Kenya: an analysis of the Kenya demographic and health survey, 2008/2009. BMC Pregnancy Childbirth..

[12] Echoka E, Makokha A, Dubourg D, Kombe Y, Nyandieka L, Byskov J (2014). Barriers to emergency obstetric care services: accounts of survivors of life threatening obstetric complications in Malindi District, Kenya. Pan Afr Med J..

[13] Byrne A, Caulfield T, Onyo P, Nyagero J, Morgan A, Nduba J (2016). (In press) Practices and perceptions of traditional and skilled birth attendants providing maternal health care for pastoralist communities in Kenya: a qualitative study. BMC Pregnancy Childbirth..

[14] Ministry of Health (2007). National Reproductive Health Policy: Enhancing Reproductive Health for all Kenyans..

[15] Izugbara C, Ezeh A, Fotso J-C (2009). The persistence and challenges of homebirths: perspectives of traditional birth attendants in urban Kenya. Health Policy Plan..

[16] Dietsch E (2010). The experience of being a traditional midwife: relationships with skilled birth attendants. Rural Remote Health..

[17] Caulfield T, Onyo P, Byrne A, Nduba J, Nyagero J, Morgan A (2015). (Submitted for review) Factors influencing place of delivery for pastoralist women in Kenya..

[18] Vieira C, Portela A, Miller T, Coast E, Leone T, Marston C (2012). Increasing the use of skilled health personnel where traditional birth attendants were providers of childbirth care: a systematic review. PLoS ONE..

[19] Tomedi A, Tucker K, Mwanthi MA (2013). A strategy to increase the number of deliveries with skilled birth attendants in Kenya. Int J Gynaecol Obstet..

[20] Dietsch E, Mulimbalimba-Masururu L (2011). Learning lessons from a traditional midwifery workforce in western Kenya. Midwifery..

[21] Pyone T, Adaji S, Madaj B, Woldetsadik T, van den Broek N (2014). Changing the role of the traditional birth attendant in Somaliland. Int J Gynaecol Obstet..

[22] Roggeveen Y, Birks L, van Kats J, Manyama M, Hatfield J, Bunders J (2013). Low utilization of skilled birth attendants in Ngorongoro Conservation Area, Tanzania: a complex reality requiring action. Health (N Y)..

[23] Kawakatsu Y, Sugishita T, Oruenjo K, Wakhule S, Kibosia K, Were E (2014). Determinants of health facility utilization for childbirth in rural western Kenya: cross-sectional study. BMC Pregnancy Childbirth..

[24] Mwaliko E, Downing R, O'Meara W, Chelagat D, Obala A, Downing T (2014). “Not too far to walk”: the influence of distance on place of delivery in a western Kenya health demographic surveillance system. BMC Health Serv Res..

[25] Anglican Overseas Aid, Mothers Union of the Anglican Church of Kenya, Nossal Institute for Global Health, Afar Pastoralist Development Association (2014). The Road Less Travelled: AACES 2013-2014 Annual Report..

[26] Amref Health Africa AMREF's position on the role and services of traditional birth attendants..

[27] O'Rourke K (1995). The effect of hospital staff training on management of obstetrical patients referred by traditional birth attendants. Int J Gynaecol Obstet..

[28] Gabrysch S, Lema C, Bedriñana E, Bautista MA, Malca R, Campbell OMR (2009). Cultural adaptation of birthing services in rural Ayacucho, Peru. Bull World Health Organ..

